# Change in suicidal ideation, depression, and anxiety following collaborative care in the community

**DOI:** 10.1186/s12875-024-02494-2

**Published:** 2024-07-05

**Authors:** Gabriela Kattan Khazanov, Courtney Benjamin Wolk, Emily Lorenc, Molly Candon, Matteo F. Pieri, David W. Oslin, Matthew J. Press, Eleanor Anderson, Emilie Famiglio, Shari Jager-Hyman

**Affiliations:** 1 Perelman School of Medicine, University of Pennsylvania, Philadelphia, PA USA; 2 Center of Excellence for Substance Addiction Treatment and Education, Corporal Michael J. Crescenz VA Medical Center, 3900 Woodland Avenue, Philadelphia, PA 19104 USA; 3 Leonard Davis Institute of Health Economics, University of Pennsylvania, Philadelphia, PA USA; 4 School of Medicine and Health Sciences, The George Washington University, Washington, DC USA; 5 Mental Illness Research Education and Clinical Center, Corporal Michael J. Crescenz VA Medical Center, Philadelphia, PA USA

**Keywords:** Collaborative care, Suicide, Suicidal ideation, Primary care, Integrated care, Community care, Depression

## Abstract

**Background:**

The Collaborative Care Model (CoCM) increases access to mental health treatment and improves outcomes among patients with mild to moderate psychopathology; however, it is unclear how effective CoCM is for patients with elevated suicide risk.

**Methods:**

We examined data from the Penn Integrated Care program, a CoCM program including an intake and referral management center plus traditional CoCM services implemented in primary care clinics within a large, diverse academic medical system. In this community setting, we examined: (1) characteristics of patients with and without suicidal ideation who initiated CoCM, (2) changes in suicidal ideation (Patient Health Questionnaire-9 [PHQ-9] item 9), depression (PHQ-9 total scores), and anxiety (Generalized Anxiety Disorder Scale-7 scores) from the first to last CoCM visit overall and across demographic subgroups, and (3) the relationship between amount of CoCM services provided and degree of symptom reduction.

**Results:**

From 2018 to 2022, 3,487 patients were referred to CoCM, initiated treatment for at least 15 days, and had completed symptom measures at the first and last visit. Patients were 74% female, 45% Black/African American, and 45% White. The percentage of patients reporting suicidal ideation declined 11%-7% from the first to last visit. Suicidal ideation severity typically improved, and very rarely worsened, during CoCM. Depression and anxiety declined significantly among patients with and without suicidal ideation and across demographic subgroups; however, the magnitude of these declines differed across race, ethnicity, and age. Patients with suicidal ideation at the start of CoCM had higher depression scores than patients without suicidal ideation at the start and end of treatment. Longer CoCM episodes were associated with greater reductions in depression severity.

**Conclusions:**

Suicidal ideation, depression, and anxiety declined following CoCM among individuals with suicidal ideation in a community setting. Findings are consistent with emerging evidence from clinical trials suggesting CoCM’s potential for increasing access to mental healthcare and improving outcomes among patients at risk for suicide.

**Supplementary Information:**

The online version contains supplementary material available at 10.1186/s12875-024-02494-2.

Support for the Collaborative Care Model (CoCM) of integrating mental health treatment in primary care has been widely documented [1, 2]. CoCM is a team-based treatment approach involving both primary care and behavioral health providers that has been shown to be cost-effective and to improve access to care and quality of life [2, 3]. Typically, CoCM focuses on treating patients with mild to moderate depression, anxiety, or alcohol misuse, and patients with more severe psychopathology, including suicidal ideation, are referred out for more intensive treatment in the community due to concerns about their appropriateness for management in the primary care context [4, 5]. Many patients in the US with more severe psychopathology, however, including those at risk for suicide, have difficulty accessing specialty mental health treatment [6].

Difficulty accessing mental health care has been linked to higher rates of suicide, which have risen 30% over the last 20 years [7, 8]. While most individuals who die by suicide are not actively engaged in mental health care, approximately two thirds interact with primary care providers in the year prior to death [9, 10]. Newer research has shown that CoCM may be a model of care that could substantially increase access to treatment and improve outcomes for individuals at risk for suicide [11]. Preliminary evidence from clinical trials demonstrates that CoCM can improve suicidal ideation [12, 13] and there is substantial support for CoCM improving overall levels of depression [12, 14]. To date, however, there is minimal research on changes in suicidal ideation following CoCM in naturalistic community settings outside of the context of clinical trials. One study that did examine this question showed that individuals with suicidal ideation received more services and had worse depression outcomes in CoCM than individuals without suicidal ideation [15]. Additionally, little is known about characteristics that differentiate CoCM patients with and without suicidal ideation, nor about the relationship between the amount of CoCM services provided and the extent of symptom reduction among individuals at risk for suicide.

We aimed to: (1) describe the characteristics of patients with and without suicidal ideation in a large academic health system; (2) examine changes in suicidal ideation, depression, and anxiety following CoCM among patients with and without suicidal ideation, and compare differences in these changes across demographic subgroups; and (3) examine the relationship between the amount of CoCM services provided and degree of symptom reduction among patients with and without suicidal ideation.

## Methods

This study was approved by the Institutional Review Board of the University of Pennsylvania, which provided a waiver of informed consent for the analysis of electronic health records (EHR). Analyses was performed in SAS Version 9.4.

### Setting and participants

The University of Pennsylvania Health System, a diverse academic medical system, launched the Penn Integrated Care (PIC) program in 2018 in eight primary care practices [5]. Since inception, PIC has since expanded to more than 35 urban and suburban primary care practices in the Penn Medicine network.

PIC augments traditional CoCM services for all patients with an intake, triage, and referral management center, referred to as the PIC Resource Center [5]. Following referral by a primary care clinician, bachelor’s level mental health intake coordinators assess patient eligibility for CoCM over the phone using standardized mental health screening measures, such as the Patient Health Questionnaire-9 (PHQ-9^17^). Patients are stratified using decision-support software [16] based on symptom severity and the presence of comorbid disorders, and are referred to the appropriate level of care, which may include self-directed resources, CoCM services in their primary care clinic, or specialty mental health care in the community. Patients who endorse suicidal ideation at the point of referral or intake are immediately assessed by a behavioral health specialist following a warm handoff. If the assessment does not indicate acute suicide risk (i.e., concern of imminent harm to self), the patient continues with the standard referral and intake process. Therefore, in the absence of acute risk, suicidal ideation does not preclude referral to CoCM services in the PIC program. Additional details about screening and triage procedures and treatment in the PIC program are discussed elsewhere [5].

The PIC CoCM team includes the mental health intake coordinators in the Resource Center, primary care clinicians, a behavioral health specialist (a master’s level mental health clinician) embedded in the primary care office, and a psychiatric consultant. Electronic health record (EHR) registries are used to identify and monitor patients [5].

## Measures

Patients completed standardized measures of depression and anxiety symptomatology at baseline (the first CoCM session) and post-treatment (last CoCM session).

The *Patient Health Questionnaire-9* (PHQ-9^17^), which includes nine items assessing depression symptoms over the previous two weeks, served as our measure of depression. The ninth item assesses suicidal ideation: “Over the last two weeks, how often have you been bothered by thoughts that you would be better off dead or of hurting yourself in some way?” We compared patients who endorsed each response option for this item (i.e., “not at all” [0 points], “several days” [1 point], “more than half the days” [2 points], or “nearly every day” [3 points]) at baseline. Scores range from 0 to 27, and acceptable reliability and validity in primary care have been established [17, 18].

The *Generalized Anxiety Disorder Scale* (GAD-7 [19]) served as our measure of anxiety. The GAD-7 assesses seven symptoms of anxiety using the same format as the PHQ-9, with scores ranging from 0 to 21. The GAD-7 has demonstrated evidence of reliability and validity in primary care settings [19, 20].

*Sociodemographic characteristics* were collected using items developed by the Department of Veteran Affairs Integrated Care program, including sex, age, and race/ethnicity [16]. Patients were presented with categories for each characteristic and self-reported the categories with which they identified.

### Analyses

We included patients with: (1) CoCM episodes lasting at least 15 days and (2) baseline and post-treatment PHQ-9 assessments, including the suicidal ideation item (i.e., item 9). We excluded patients with episodes limited to referral management and those with only mild depression symptoms (i.e., PHQ-9 total score depression *≤* 5 [21]) unless they endorsed suicidal ideation (sTable 1).

To describe characteristics of patients with and without suicidal ideation (Aim 1), we compared patients reporting no suicidal ideation (0 score) to those reporting any level of suicidal ideation (1–3) on the ninth PHQ-9 item at baseline. All subsequent analyses were run separately for each group. For Aim 2, we compared baseline and post-treatment PHQ-9 and GAD-7 scores using pairwise t-tests for each group overall and for individuals in each demographic subgroup. We then reran analyses comparing baseline and post-treatment PHQ-9 scores among patients who endorsed a 1 versus 2 versus 3 on the PHQ-9 suicidal ideation item; we also reran analyses after subtracting the suicidal ideation item from the PHQ-9 total score to account for differences in scores due to the inclusion of that item. We estimated confidence intervals to compare differences between baseline and post-treatment PHQ-9 scores based on baseline reports of suicidal ideation. Subsequently, we used ANOVAs with post-hoc Tukey tests to compare differences in PHQ-9 and GAD-7 reductions across demographic subgroups. For Aim 3, we examined the relationship between CoCM episode length (less than 1 month, 1–3 months, 4–5 months, and 6 or more months) and reductions in PHQ-9 scores for patients with and without suicidal ideation using confidence intervals. We reran these analyses substituting the number of CoCM encounters, divided into quartiles of the distribution of the number of encounters, for CoCM episode length.

## Results

### Characteristics of patients included in the study

**Fig. 1:**
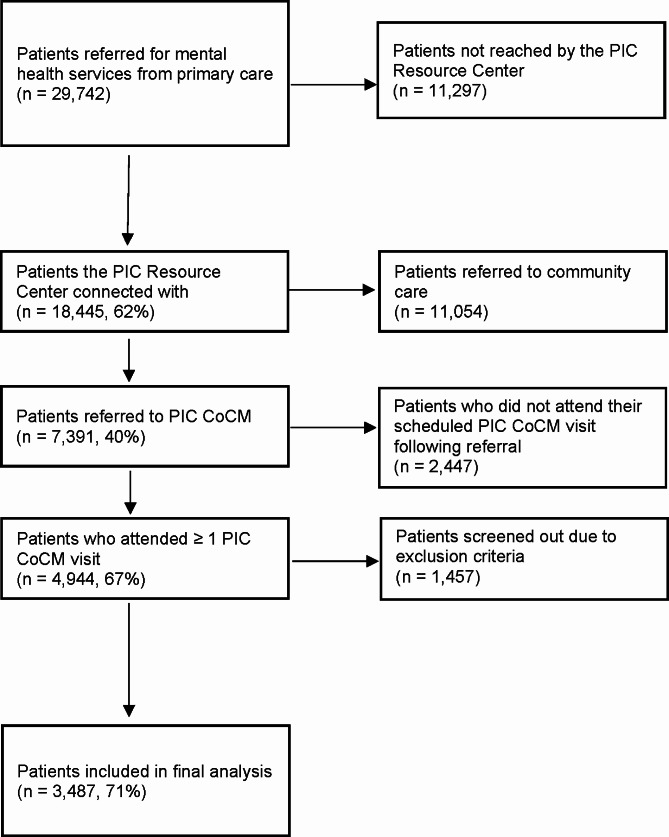
CONSORT diagram of participants included in analyses. Figure 1 caption. PIC = Penn Integrated Care. CoCM = Collaborative Care Model

A total of 29,742 patients 18 years of age or older were referred to the PIC Resource Center for an intake assessment between 2018 and 2022; 3,487 initiated PIC CoCM services in their primary care practices and were included in analyses (Fig. 1). We included all patients with CoCM episodes lasting at least 15 days who had more than one PHQ-9 score (enabling evaluation of changes in outcomes from baseline to post-treatment). While we cannot evaluate changes in outcomes for patients with only baseline PHQ-9s, we compared baseline scores for those included and excluded from the study sample (sTable 1 – categories were not mutually exclusive).

We found slightly, although non-significantly, higher baseline PHQ-9 scores for patients only in referral management (*n* = 754; M = 11.38) and those with CoCM episodes lasting less than 15 days (*n* = 304; M = 11.75) compared to the study sample (M = 11.00). These slight differences may be due to patients with more severe symptoms being referred to specialty mental healthcare immediately (referral management) or after 1–2 CoCM sessions (episodes lasting less than 15 days). We found significantly lower baseline PHQ-9 scores for patients in an active episode (i.e., currently engaged in ongoing CoCM services) during data collection (*n* = 429; M = 9.20) and those with PHQ-9 scores less than 5 with no suicidal ideation (*n* = 769, M = 2.65) compared to the study sample (M = 11.00). As thresholds for CoCM referrals changed depending on staff availability, this threshold may have been lower at the time of data collection than in prior time periods.

### Characteristics of patients with and without suicidal ideation

Of patients included in the study (*N* = 3,487), 368 (11%) reported suicidal ideation on the PHQ-9 item 9 at baseline. These patients were mostly female (68%) and between the ages of 18–44 (65%; Table 1). About half identified as Black (50%), with many identifying as White (37%) or Other (11%)^1^; 6% identified as Hispanic or Latinx. Patients who did not report suicidal ideation were also mostly female (74%) and between the ages of 18–44 (57%). About half identified as Black (45%) and White (45%), with fewer identifying as another race (7%); 4% also identified as Hispanic or Latinx.

**Table 1:** Mean differences in PHQ-9 scores by sociodemographic characteristics and the presence of suicidal ideation at baseline

	Suicidal Ideation					No Suicidal Ideation				
	N	%	First PHQ	Last PHQ	Difference	N	%	First PHQ	Last PHQ	Difference
Overall	368	-	15.62	11.01	4.61**	3119	-	10.45	6.18	4.27**
Sex										
Female	250	68	15.92	11.21	4.71**	2322	74	10.55	6.19	4.36**
Male	118	32	14.99	10.59	4.40**	797	26	10.17	6.15	4.02**
ANOVA	0.19					3.76*				
Race										
Asian	8	2	16.88	9.38	7.50**	91	3	10.2	5.52	4.68**
Black	184	50	16.12	11.76	4.36**	1390	45	11.14	6.6	4.54**
White	135	37	14.31	9.87	4.44**	1418	45	9.8	5.74	4.06**
Other Race^a^	41	11	17.44	11.76	5.68**	220	7	10.44	6.72	3.72**
ANOVA	1.1							4.50**		
								Black and White**		
								Black and Other Race**		
Ethnicity										
Hispanic/Latinx	21	6	16.62	9.33	7.29**	126	4	10.58	6.34	4.24**
Non-Hispanic/Latinx	347	94	15.56	11.12	4.44**	2993	96	10.45	6.18	4.27**
ANOVA	4.12**					0.01				
Age										
Under 24	62	17	15.92	10.24	5.68**	277	9	10.63	6.43	4.20**
25 to 34	98	27	15.32	10.45	4.87**	776	25	10.44	6.53	3.91**
35 to 44	79	21	15.43	10.89	4.54**	701	23	10.45	6.31	4.14**
45 to 54	48	13	17.08	12.38	4.70**	467	15	11.09	6.46	4.63**
55 to 64	40	11	16.25	13.83	2.42**	389	12	10.98	6.35	4.63**
65 to 74	25	7	14.6	10.24	4.36**	347	11	9.65	5.06	4.59**
75+	16	4	12.88	8.19	4.69**	162	5	8.88	4.78	4.10**
ANOVA	1.16						2.38**			
							25 to 34 and 45 to 54*			
							25 to 34 and 55 to 64*			

Note. ANOVAs tested differences between demographic subgroups among patients with and without suicidal ideation at baseline and were followed up with Tukey post-hoc tests. Included numbers represent the F statistic for each ANOVA.

PHQ-9 = Patient Health Questionnaire – 9 items, a measure of depression severity. ANOVA = analysis of variance

^a^Groups with small proportions of patients were collapsed into the “Other” category to facilitate analyses – this category included American Indian or Alaskan Native, Native Hawaiian or Pacific Islander, Other Race, and Patient Declined/Unknown.

*p < .10, **p < .05

### Changes in suicidal thoughts, depression, and anxiety following CoCM

Among the 368 patients reporting suicidal ideation at baseline, most experienced ideation for several days (*n* = 256, 69%; sTable 2) versus more than half the days (*n* = 68; 19%) or nearly every day (*n* = 44; 12%). Most showed improvement in suicidal ideation post-treatment (*n* = 193; 52%), while almost half showed no change (*n* = 169; 46%). Very few showed worsening of ideation (*n* = 6; 2%; sTable 3). When considering patients who showed no suicidal ideation at baseline (*n* = 3119), a negligible number (*n* = 42; 1%) showed some level of suicidal ideation at post-treatment (sTable 3). Across the full sample, the number of patients reporting suicidal ideation of any severity decreased by 37% from baseline to post-treatment (11% vs. 7%). Among the 7% of patients reporting suicidal ideation at post-treatment (*n* = 231), most reported experiencing ideation for several days (64%; *n* = 149), with some reporting experiencing ideation more than half the days (21%, *n* = 48) or nearly every day (15%, *n* = 34).

**Fig. 2:**
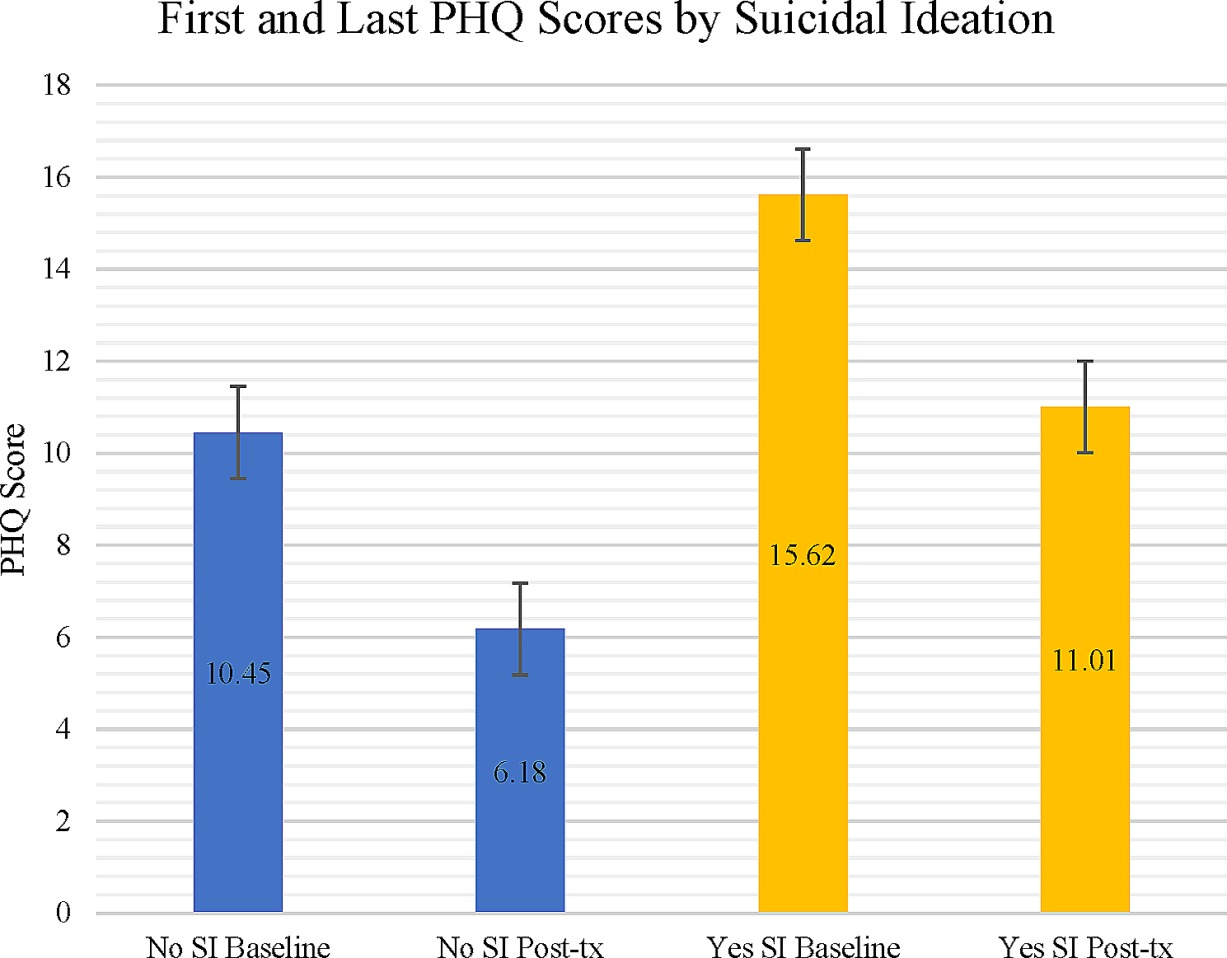
First and last PHQ-9 scores by the presence of suicidal ideation at baseline and post-treatment. Figure 2 caption. PHQ-9 = Patient Health Questionnaire – 9 items

For patients with suicidal ideation, depression severity declined by an average of 4.61 PHQ-9 total score points, from 15.62 (moderately severe) to 11.01 (moderate; *p* < .005). These reductions were statistically significant across demographic subgroups (Table 1; Fig. 2), levels of ideation (sTable 2), and when excluding the suicidal ideation item from total PHQ-9 scores (sTable 4). Patients with lower rather than higher levels of suicidal ideation experienced greater, albeit not statistically significant, reductions in depression (sTable 2). For patients without baseline suicidal ideation, depression severity declined by 4.27 points, from 10.45 (moderate) to 6.18 (mild); again, all demographic subgroups experienced a significant decline. Patients with baseline suicidal ideation scored significantly higher on the PHQ-9 across timepoints than patients without baseline suicidal ideation (sTable 5).

We found significant differences in declines in depression severity across race, ethnicity, and age for individuals with and without baseline suicidal ideation (Table 1, ANOVAs). Among those who reported suicidal ideation at baseline, we found differences in declines in depression severity depending on ethnicity (higher for Hispanic/Latinx patients – 7.29 versus 4.44; Table 1). Among those not reporting suicidal ideation at baseline, we found differences in declines in depression severity depending on race (higher among individuals identifying as Black (4.54) versus White (4.06) and as Black versus another race (3.72)) and age (marginally lower for 25–34 year olds (3.91) versus 45–54 year olds (4.63) and 55–64 year olds (4.63)), with marginal differences depending on sex (marginally higher declines for females (4.36) than males (4.02)).

These results replicated for anxiety severity (Table 2). For patients with baseline suicidal ideation, anxiety severity (GAD-7 total scores) declined by 3.43 points, from 12.74 (moderate) to 9.31 (mild; *p* < .005), while anxiety severity declined by 3.92 points, from 10.14 (moderate) to 6.22 (mild; *p* < .005) for those without baseline ideation; all demographic subgroups experienced significant declines. Anxiety scores differed depending on ethnicity among those who reported suicidal ideation at baseline (higher for Hispanic/Latinx patients (5.75) than non Hispanic/Latinx patients (3.28)); no differences were found among those who did not report suicidal ideation at baseline.

**Table 2:** Mean differences in GAD-7 scores by sociodemographic characteristics and the presence of suicidal ideation at baseline

	Suicidal Ideation					No Suicidal Ideation				
	N	%	First GAD	Last GAD	Difference	N	%	First GAD	Last GAD	Difference
Overall	336	-	12.74	9.31	3.43**	3100	-	10.14	6.22	3.92**
Sex										
Female	230	68	13.25	9.77	3.48**	2307	74	10.29	6.32	3.97**
Male	106	32	11.61	8.31	3.30**	793	26	9.69	5.92	3.77**
ANOVA	0.1					1.21				
Race										
Asian	6	2	10.67	5	5.67**	91	3	9.1	5.53	3.57**
Black	166	49	12.95	9.89	3.06**	1378	44	10.45	6.63	3.82**
White	126	38	12.4	8.68	3.72**	1414	46	9.82	5.72	4.10**
Other Race^a^	38	11	13.26	9.55	3.71**	217	7	10.66	7.19	3.47**
ANOVA	0.94					2.05				
Ethnicity										
Hispanic/Latinx	20	6	14.4	8.65	5.75**	125	4	10.82	6.8	4.02**
Non-Hispanic/Latinx	316	94	12.63	9.35	3.28**	2975	96	10.11	6.19	3.92**
ANOVA	4.97**					0.07				
Age										
Under 24	58	17	11.98	9.1	2.88**	276	9	10.55	6.41	4.14**
25 to 34	90	27	13.3	9.24	4.06**	774	25	10.57	6.52	4.05**
35 to 44	72	21	12.58	9.1	3.48**	696	23	10.75	6.64	4.11**
45 to 54	44	13	14.32	11.22	3.10**	466	15	10.41	6.58	3.83**
55 to 64	33	10	13.21	9.79	3.42**	385	12	9.99	6.36	3.63**
65 to 74	25	8	12	9.16	2.84**	343	11	8.52	4.7	3.82**
75+	14	4	8.21	4.79	3.42**	160	5	7.75	4.48	3.27**
ANOVA	0.47					1.37				

Note. ANOVAs tested differences between demographic subgroups among patients with and without suicidal ideation at baseline. Included numbers represent the F statistic for each test

GAD-7 = Generalized Anxiety Disorder – 7 item questionnaire (a measure of generalized anxiety). ANOVA = analysis of variance

^a^Groups with small proportions of patients were collapsed into the “Other” category to facilitate analyses – this category included American Indian or Alaskan Native, Native Hawaiian or Pacific Islander, Other Race, and Patient Declined/Unknown.

*p < .10, **p < .05

### Relationship between CoCM and symptom reduction

Patients receiving CoCM for longer periods of time showed significantly greater declines in PHQ-9 scores; this pattern held both for patients who did and did not endorse suicidal ideation at baseline, but not for patients treated in CoCM for over six months (Table 3). These results replicated when examining number of CoCM encounters instead of treatment length (sTable 6).

**Table 3:** PHQ-9 mean differences by episode length and the presence of suicidal ideation at baseline

						95% CI of difference	
	Episode Length	N	First PHQ	Last PHQ	Diff PHQ	LL	UL
No elevations in suicidal ideation at baseline	< 1 month	241	10.83	8.22	2.61	2.06	3.15
	1 to 3 months	1609	10.45	6.62	3.83	3.63	4.04
	4 to 5 months	1027	10.29	5.12	5.17	4.92	5.43
	6 + months	242	10.77	5.75	5.02	4.45	5.59
Elevated suicidal ideation at baseline	< 1 month	88	16.97	15.6	1.37	0.38	2.35
	1 to 3 months	170	15.36	10.61	4.75	3.83	5.66
	4 to 5 months	85	14.74	7.41	7.33	6.11	8.54
	6 + months	25	15.64	9.84	5.8	2.02	9.58

Note. PHQ = Patient Health Questionnaire – 9 items, a measure of depression. CI = confidence intervals,

LL = lower limit, UL = upper limit

## Discussion

In a diverse, urban academic health system, 11% of patients engaged in primary-care-based CoCM reported experiencing suicidal ideation at baseline, most at mild levels, which reduced to 7% following CoCM. Suicidal ideation improved following CoCM for most patients and very rarely worsened. Depression and anxiety declined significantly both among patients who did and did not report suicidal ideation at baseline; this result held for all demographic subgroups and across sensitivity analyses. Declines in depression and anxiety severity did, however, differ across ethnicity, race, and age among patients with and without suicidal ideation at baseline. Additionally, patients with baseline suicidal ideation had higher depression scores than patients without baseline suicidal ideation across timepoints. Among both patient groups, length of time in CoCM and number of sessions were each associated with greater declines in depression severity, except when patients were treated for over six months.

These findings demonstrate that suicidal ideation declines following ongoing CoCM services in a naturalistic community setting, and that depression and anxiety severity also decline following CoCM among individuals at risk for suicide across multiple demographic subgroups. These results expand upon emerging evidence from clinical trials showing that CoCM can lead to symptom reduction in patients with non-acute, but elevated suicide risk [12]. Together, findings indicate that CoCM, which increases access to mental health care and improves outcomes, may be an appropriate treatment modality for individuals with non-acute suicidal ideation. Additionally, a strength of CoCM is that is affords access to behavioral health specialists that can conduct suicide risk assessments and support patients with greater acuity in connecting to higher levels of care when clinically indicated. Treating individuals at risk for suicide in CoCM has the potential to reduce rates of suicide nationwide, given that most patients who die by suicide interact with primary care, but not specialty mental health care, in the year prior to their deaths [9]. Referral and triage systems that provide more immediate services to patients at acute risk for suicide seen in primary care settings can help balance the need for suicide risk management with the increased access to care that CoCM provides.

Importantly, we found that among patients with suicidal ideation at baseline, those identifying as Hispanic/Latinx versus not Hispanic/Latinx had greater declines in depression and anxiety severity. Additionally, among patients without suicidal ideation at baseline, those identifying as Black versus White or another race had significantly greater declines in depression severity. These findings complement a burgeoning literature showing that patients identifying as racial or ethnic minorities, including those identifying as Black or Latinx, show improved access to mental health care and clinical outcomes in CoCM [22, 23]. A recent review concluded that the evidence supporting the effectiveness of CoCM for mental health treatment among patients identifying as racial or ethnic minorities is larger than for any other intervention [22]. Altogether, these findings demonstrate that CoCM may be particularly effective for patients in minoritized groups for a variety of reasons, including that it reduces barriers to initiating mental health treatment by affording access to a range of providers in a single setting and is perceived as convenient and private (i.e., less stigmatizing) by patients [22]. While we also found that older versus younger individuals and patients identifying as females versus males had greater declines in depression severity, these differences were marginally significant.

Our finding that longer courses of CoCM and more treatment sessions reduced depression to a greater extent among individuals with and without suicidal ideation is consistent with previous studies showing an association between CoCM treatment length and reduced symptomatology [24, 25]. We also found that depression scores did not continue to decrease for patients seen in CoCM over six months. Previous research has demonstrated that the minimum effective dose for CoCM is around 4–5 sessions [26]; combined with this research, our findings suggest that an optimal course of CoCM may consist of between 4 and 8 sessions (or around 2–4 months), but that care lasting beyond six months may not add value.

We also found that patients reporting suicidal ideation had higher depression scores at both their first and last treatment sessions relative to patients without suicidal ideation, similar to a prior study demonstrating worse depression outcomes among CoCM patients with versus without suicidal ideation [15]. These findings suggest that the presence of suicidal ideation in CoCM patients may be an indicator of higher levels of psychopathology and the need for additional services. They also highlight the benefit of using CoCM as part of a stepped-care model, and suggest that it may be appropriate to refer patients to specialty mental health care after around six months of CoCM care.

This study had several limitations. First, as this was a study of CoCM in a naturalistic community setting, we did not have a control group that did not receive CoCM services. Therefore, patients’ symptoms may have improved due to time versus the provision of CoCM services. Second, as most patients had measures of depression and anxiety only from their first and last treatment sessions and as measures from intermediate sessions were not accessible in the EHR, we were unable to conduct longitudinal analyses. Third, we were unable to compare changes in outcomes among patients who had both baseline and post-treatment PHQ-9s to those without post-treatment scores. Additionally, analyses comparing baseline PHQ-9 scores indicated significant differences among patients excluded from the study due to being in an active CoCM episode at the time of data collection versus included patients. Fourth, given the constraints of collecting data in a naturalistic treatment setting, we relied on patients’ self-reported symptoms of suicidal ideation, depression, and anxiety instead of clinician-rated measures. Relatedly, we used Item 9 of the PHQ-9 to measure suicidal ideation; although this item is widely used to measure suicidal ideation in treatment settings and predicts suicidal behavior [27, 28], a longer, multi-item measure would have been preferred. Relatedly, our use of one item to measure suicidal ideation may have made it more difficult to detect changes given relatively low overall scores. Importantly, this limitation is common across studies focusing on non-acute patients, such as those typically seen in primary care settings. Lastly, given our interest in studying change following CoCM, we only included patients referred to CoCM. As patients with more severe psychopathology were generally referred to community care, we do not know whether our findings extend to these patients.

## Conclusions

In this study, we demonstrated that suicidal ideation can be treated in CoCM. Suicidal ideation decreased following CoCM, and depression and anxiety declined among both patients with and without suicidal ideation at baseline. Furthermore, additional time spent in CoCM was associated with greater declines in depression severity. Including patients with suicidal ideation in CoCM has the potential to substantially increase access to mental health care and improve outcomes for many individuals without current access to care. Future research, including controlled clinical trials using multi-item measures of suicidal ideation, can complement the present study by examining whether CoCM reduces suicidal ideation and other outcomes, particularly among patients at higher risk for suicide.

### Electronic supplementary material

Below is the link to the electronic supplementary material.

Supplementary Material 1

## Data Availability

Our data source is medical records, which are not publicly accessible. The SAS code used to generate findings is available upon request to the corresponding author at gabriela.khazanov@va.gov, 3900 Woodland Avenue, Philadelphia, PA 19104.

## Footnotes

1 Groups with small proportions of patients were collapsed into the “Other” category to facilitate analyses – this category included American Indian or Alaskan Native, Native Hawaiian or Pacific Islander, Other Race, and Patient Declined/Unknown.

## Abbreviations

CoCM: Collaborative Care Model
EHR: Electronic Health Record
GAD-7: Generalized Anxiety Disorder Scale – 7 items
PHQ-9: Patient Health Questionnaire – 9 items
PIC: Penn Integrated Care
