# Supplementary material for: Change in suicidal ideation, depression, and anxiety following collaborative care in the community
Source: BMC Prim Care. 2024 Jul 5;25:241. doi: 10.1186/s12875-024-02494-2 (PMC11225270; doi:10.1186/s12875-024-02494-2)
Supplement: Supplementary file 1 — Supplementary Material 1 [file 12875_2024_2494_MOESM1_ESM.docx]

**Supplemental Table 1**

*PHQ-9 scores for individuals included and excluded from the final study sample*

| Group | PHQ-9 at Initial Visit | 95% CI | SI score breakdown | Item 9 (SI score) at Initial Visit | Number of Patients |
| --- | --- | --- | --- | --- | --- |
| Incomplete baseline or follow-up data | 11.50 | (-134.60, 157.60) | 1: 0 - not at all 1: 1 - Several days | 0.50 | 2 |
| In active episode at time of data collection | 9.20 | (8.72, 9.69) | 381 : 0 - Not at all 42: 1 - Several days 6: 2- More than half the days 1: 3 - Nearly every day | 0.13 | 429 |
| Collaborative Care episode lasted less than 15 days | 11.75 | (10.98, 12.52) | 228: 0 - Not at all 51: 1 - Several days 26: 2- More than half the days 22: 3 - Nearly every day | 0.52 | 304 |
| Referral management | 11.38 | (10.89, 11.86) | 622: 0 - Not at all 107: 1 - Several days 19: 2- More than half the days 17: 3 - Nearly every day | 0.26 | 754 |
| PHQ-9 was less than 5 and no SI was reported | 2.65 | (2.56, 2.74) | 859: 0 - Not at all | 0 | 769 |
| Study sample (data presented in paper) | 11.00 | (10.85, 11.15) | 3,119: 0 - Not at all 256: 1 - Several days 68: 2- More than half the days 44: 3 - Nearly every day | 0.15 | 3,487 |

*Note.* PHQ = Patient Health Questionnaire – 9 items, a measure of depression. SI = Suicidal ideation. Suicidal ideation was measured with Item 9 on the PHQ-9 (“thoughts that you would be better off dead, or of hurting yourself” over the previous two weeks). The categories included in this table are not mutually exclusive (i.e., some patients were included in more than one category).

**Supplemental Table 2**

*Reductions in PHQ-9 scores based on level of suicidal ideation at baseline*

| SI Level (first PHQ) |  |  |  |  |  |  |  |  |
| --- | --- | --- | --- | --- | --- | --- | --- | --- |
|  | N | % | First PHQ | Last PHQ | Difference | P-Value | 95% CI of difference | |
|  |  |  |  |  |  |  | LL | UL |
| 0 (No SI) | 3119 | - | 10.45 | 6.18 | 4.27 | <0.0001 | 4.12 | 4.33 |
| 1 (SI “several days”) | 256 | 69 | 14.04 | 8.86 | 5.18 | <0.0001 | 4.43 | 5.91 |
| 2 (“more than half the days”) | 68 | 19 | 18.10 | 14.72 | 3.38 | <0.0001 | 1.94 | 4.82 |
| 3 (“nearly every day”) | 44 | 12 | 21.00 | 17.80 | 3.20 | 0.0083 | 0.87 | 5.54 |

*Note.* PHQ = Patient Health Questionnaire – 9 items, a measure of depression. SI = Suicidal ideation. Suicidal ideation was measured with Item 9 on the PHQ-9 (“thoughts that you would be better off dead, or of hurting yourself” over the previous two weeks).

**Supplemental Table 3**

*Changes in suicidal ideation at baseline and post-treatment*

|  | Post-treatment suicidal ideation | | | |
| --- | --- | --- | --- | --- |
| Baseline suicidal ideation | 0 | 1 | 2 | 3 |
| 0 | 3077 | 39 | 2 | 1 |
| 1 | 154 | 97 | 5 | 0 |
| 2 | 18 | 9 | 40 | 1 |
| 3 | 7 | 4 | 1 | 32 |

*Note*. Scores are drawn from the Patient Health Questionnaire – 9 items (PHQ-9) Item 9 (“Over the last two weeks, how often have you been bothered by thoughts that you were better off dead or of hurting yourself in some way?”; 0 = “not at all”, 1 = “several days”, 2 = “more than half the days”, 3 = “nearly every day”). Highlighted cells represent numbers of patients in each category. Dark grey represents a decrease in suicidal ideation level, white represents no change in suicidal ideation level, and light grey represents an increase in suicidal ideation level.

**Supplemental Table 4**

*Mean differences in PHQ-9 scores by the presence of suicidal ideation at baseline, adjusted to account for the contribution of the suicidal ideation item (item 9)*

| Suicidal Ideation | N | First PHQ | Last PHQ | Difference | P-Value | 95% CI of difference | |
| --- | --- | --- | --- | --- | --- | --- | --- |
|  |  |  |  |  |  | LL | UL |
| No | 3119 | 10.45 | 6.17 | 4.28 | <0.0001 | 4.13 | 4.44 |
| Yes | 368 | 14.2 | 10.2 | 4 | <0.0001 | 3.4 | 4.6 |

*Note.* PHQ = Patient Health Questionnaire – 9 items, a measure of depression. CI = confidence intervals. In this analysis, we subtracted PHQ-9 item 9 (the suicidal ideation item) from the overall PHQ-9 score to account for differences in scores due to the inclusion of this item.

**Supplemental Table 5**

*Baseline and final PHQ-9 scores by the presence of suicidal ideation at baseline*

|  | Suicidal Ideation at Baseline | | | | | | | No Suicidal Ideation at Baseline | | | | | | |
| --- | --- | --- | --- | --- | --- | --- | --- | --- | --- | --- | --- | --- | --- | --- |
|  | N | First PHQ | 95% CI on first | | Last PHQ | 95% CI on last | | N | First PHQ | 95% CI on first | | Last PHQ | 95% CI on last | |
|  |  |  | LL | UL |  | LL | UL |  |  | LL | UL |  | LL | UL |
| Overall | 368 | 15.62 | 15.07 | 16.17 | 11.01 | 10.26 | 11.77 | 3119 | 10.45 | 10.32 | 10.59 | 6.18 | 6.02 | 6.34 |
| Sex | | | | | | | | | | | | | | |
| Female | 250 | 15.92 | 15.26 | 16.58 | 11.21 | 10.33 | 12.09 | 2322 | 10.55 | 10.39 | 10.71 | 6.19 | 6.01 | 6.37 |
| Male | 118 | 14.99 | 13.99 | 16 | 10.59 | 9.13 | 12.05 | 797 | 10.17 | 9.9 | 10.44 | 6.15 | 5.84 | 6.47 |
| Race | | | | | | | | | | | | | | |
| Asian | 8 | 16.88 | 13.52 | 20.23 | 9.38 | 3.9 | 14.85 | 91 | 10.2 | 9.44 | 10.95 | 5.52 | 4.67 | 6.36 |
| Black | 184 | 16.12 | 15.31 | 16.93 | 11.76 | 10.64 | 12.89 | 1390 | 11.14 | 10.93 | 11.36 | 6.6 | 6.34 | 6.85 |
| White | 135 | 14.31 | 13.49 | 15.13 | 9.87 | 8.9 | 11.04 | 1418 | 9.8 | 9.61 | 9.99 | 5.74 | 5.52 | 5.95 |
| Other Race^a^ | 41 | 17.44 | 15.64 | 19.24 | 11.76 | 9.5 | 14.01 | 220 | 10.44 | 9.92 | 10.96 | 6.72 | 6.14 | 7.29 |
| Ethnicity | | | | | | | | | | | | | | |
| Hispanic/Latinx | 21 | 16.62 | 14.18 | 19.06 | 9.33 | 5.66 | 13.01 | 126 | 10.58 | 9.85 | 11.31 | 6.34 | 5.61 | 7.07 |
| Non-Hispanic/Latinx | 347 | 15.56 | 14.99 | 16.13 | 11.12 | 10.34 | 11.89 | 2993 | 10.45 | 10.31 | 10.59 | 6.18 | 6.01 | 6.34 |
| Age | | | | | | | | | | | | | | |
| Under 24 | 62 | 15.92 | 14.59 | 17.24 | 10.24 | 8.35 | 12.14 | 277 | 10.63 | 10.19 | 11.07 | 6.43 | 5.95 | 6.91 |
| 25 to 34 | 98 | 15.32 | 14.24 | 16.4 | 10.45 | 9.01 | 11.88 | 776 | 10.44 | 10.16 | 10.71 | 6.53 | 6.22 | 6.84 |
| 35 to 44 | 79 | 15.43 | 14.31 | 16.55 | 10.89 | 9.37 | 12.4 | 701 | 10.45 | 10.16 | 10.74 | 6.31 | 5.97 | 6.64 |
| 45 to 54 | 48 | 17.08 | 15.53 | 18.63 | 12.38 | 10.11 | 14.64 | 467 | 11.09 | 10.7 | 11.47 | 6.46 | 6.01 | 6.92 |
| 55 to 64 | 40 | 16.25 | 14.31 | 18.19 | 13.83 | 11.24 | 16.41 | 389 | 10.98 | 10.59 | 11.38 | 6.35 | 5.9 | 6.8 |
| 65 to 74 | 25 | 14.6 | 12.66 | 16.54 | 10.24 | 7.58 | 12.9 | 347 | 9.65 | 9.28 | 10.03 | 5.06 | 4.62 | 5.49 |

*Note.* PHQ = Patient Health Questionnaire – 9 items, a measure of depression. CI = confidence intervals, LL = lower limit, UL = upper limit.

^a^Groups with small proportions of patients were collapsed into the “Other” category to facilitate analyses – this category included American Indian or Alaskan Native, Native Hawaiian or Pacific Islander, Other Race, and Patient Declined/Unknown.

**Supplemental Table 6**

*PHQ-9 mean differences by number of encounters and the presence of suicidal ideation at baseline*

|  | | | | | | 95% CI of difference | |
| --- | --- | --- | --- | --- | --- | --- | --- |
|  | Quartile | N | First PHQ | Last PHQ | Diff PHQ | LL | UL |
| No elevations in suicidal ideation at baseline |  |  |  |  |  |  |  |
|  | 1 (0-1) | 826 | 10.65 | 7.49 | 3.16 | 2.86 | 3.46 |
|  | 2 (2-3) | 701 | 10.45 | 6.98 | 3.47 | 3.18 | 3.76 |
|  | 3 (4-7) | 959 | 10.26 | 5.32 | 4.94 | 4.67 | 5.2 |
|  | 4 (8+) | 633 | 10.5 | 4.9 | 5.6 | 5.26 | 5.94 |
| Elevated suicidal ideation at baseline |  |  |  |  |  |  |  |
|  | 1 (0-1) | 171 | 16.58 | 13.85 | 2.73 | 1.78 | 3.67 |
|  | 2 (2-3) | 69 | 15.16 | 9.94 | 5.22 | 3.84 | 6.6 |
|  | 3 (4-7) | 79 | 14.59 | 7.91 | 6.68 | 5.33 | 8.04 |
|  | 4 (8+) | 49 | 14.57 | 7.61 | 6.96 | 5.41 | 8.51 |

*Note.* Quartiles were estimated by dividing the distribution of the number of encounters into four.

PHQ = Patient Health Questionnaire – 9 items, a measure of depression. CI = confidence intervals, LL = lower limit, UL = upper limit.
